# Impact of fludarabine and treosulfan on ovarian tumor cells and mesothelin chimeric antigen receptor T cells

**DOI:** 10.1007/s00262-024-03740-3

**Published:** 2024-07-02

**Authors:** Ibrahim El-Serafi, Isabella Micallef Nilsson, Alina Moter, Zhe Duan, Jonas Mattsson, Isabelle Magalhaes

**Affiliations:** 1 Department of Oncology-Pathology, Karolinska Institutet, Stockholm, Sweden; 2 Basic Medical Sciences Department, College of Medicine, Ajman University, Ajman, UAE; 3 Gloria and Seymour Epstein Chair in Cell Therapy and Transplantation, Princess Margaret Cancer Centre and University of Toronto, Princess Margaret Cancer Centre, University Health Network, Toronto, ON Canada; 4 Department of Immunology and Transfusion Medicine, Karolinska University Hospital, Stockholm, Sweden

**Keywords:** Fludarabine, Treosulfan, Mesothelin, CAR T cell, Ovarian cancer

## Abstract

In addition to their immunosuppressive effect, cytostatics conditioning prior to adoptive therapy such as chimeric antigen receptor (CAR) T cells may play a role in debulking and remodeling the tumor microenvironment. We investigated in vitro the killing efficacy and impact of treosulfan and fludarabine on ovarian cancer cells expressing mesothelin (MSLN) and effect on MSLN-targeting CAR T cells. Treosulfan and fludarabine had a synergetic effect on killing of SKOV3 and OVCAR4 cells. Sensitivity to the combination of treosulfan and fludarabine was increased when SKOV3 cells expressed MSLN and when OVCAR4 cells were tested in hypoxia, while MSLN cells surface expression by SKOV3 and OVCAR4 cells was not altered after treosulfan or fludarabine exposure. Exposure to treosulfan or fludarabine (10 µM) neither impacted MSLN-CAR T cells degranulation, cytokines production upon challenge with MSLN + OVCAR3 cells, nor induced mitochondrial defects. Combination of treosulfan and fludarabine decreased MSLN-CAR T cells anti-tumor killing in normoxia but not hypoxia. In conclusion, treosulfan and fludarabine killed MSLN + ovarian cancer cells without altering MSLN-CAR T cells functions (at low cytostatics concentration) even in hypoxic conditions, and our data support the use of treosulfan and fludarabine as conditioning drugs prior to MSLN-CAR T cell therapy.

**Supplementary Information:**

The online version contains supplementary material available at 10.1007/s00262-024-03740-3.

## Introduction

Epithelial ovarian cancer is a gynecologic malignancy with very poor prognosis [1]. New therapies are urgently needed since, despite extensive surgery and chemotherapy, up to 50% of patients suffer from disease relapse.

Adoptive cell therapy using genetically engineered T cells expressing chimeric antigen receptor (CAR) represent a major breakthrough in cancer treatment but with limited efficacy against solid tumors [2]. Most solid tumors contain hypoxic regions representing an inhospitable environment for T cells, contributing to CAR T cell treatments. Mesothelin (MSLN) is a surface glycoprotein with low expression on normal cells and increased expression in many solid tumors including ovarian cancer [3]. Several MSLN-associated therapies exist such as MSLN DNA vaccine [4], therapeutic agent biding to MSLN [5], and MSLN-targeting CAR T cells [3].

Cytostatics conditioning prior to CAR T cell therapy plays an important role in hematological malignancies treatment [6] through several mechanisms: by increasing CAR T cell expansion and persistence [7], sensitizing tumor cells to immunotherapy, and inhibiting suppressive immune cells [8]. Cyclophosphamide and/or fludarabine (nucleoside analogs) cytostatics are the golden standard conditioning drugs prior to CD19 CAR therapy [9]. Treosulfan (alkylating agent) has been previously used in treating ovarian cancer [10] and conditioning prior to hematopoietic stem cell transplantation (HSCT) [11] but not yet tested prior to CAR T cell therapy*.* Treosulfan and fludarabine combination is commonly used and shown to improve HSCT treatment efficacy and reduce related toxicities [12, 13].

In this study, we investigated treosulfan and fludarabine anti-tumor efficacy against ovarian cell lines, impact of MSLN expression and hypoxia, and effect on MSLN-CAR T cells.

## Materials and methods

### Cytostatics

Treosulfan (Sigma-Aldrich), a water-soluble prodrug that is activated non-enzymatically, was preincubated at 37 °C for 24 h to achieve 100% activation [14]. Fludarabine phosphate was a gift from Prof. Moustapha Hassan (Karolinska Institutet, Sweden).

### Cells and cell culture

SKOV3 cells (HTB-77, ATCC), OVCAR3 (HTB-161, ATCC), and OVCAR4 cells (gift from Prof. Kaisa Lehti, Karolinska Institutet) were transduced (subsequently referred to as MSLN+) using MSLN-encoding retroviral vectors to obtain high and stable MSLN cell expression [15].

Peripheral blood mononuclear cells (PBMCs) isolated from healthy donor’s (HDs) buffy coats (Karolinska University Hospital, Huddinge, Sweden) were used to produce MSLN-CAR T cells [15]. Three different MSLN-CAR constructs (kindly provided by Prof. M. Sadelain, Memorial Sloan Kettering Cancer Center, New York, USA) differing in their intracellular domain were used: CD28–CD3*ζ* (M28z), 4-1BB–CD3*ζ* (MBBz), and CD28–ITAM2–ITAM3-mutated CD3*ζ* (M1XX) [15].

### Cytostatics anti-tumor efficacy

Parental and MSLN + OVCAR4 and SKOV3 cells were incubated with treosulfan (1–10,000 µM) or fludarabine (1–1000 µM) for 48 h at 21% or 2% O_2_. Each cytostatic concentration was used in triplicates, and experiments were repeated 3–5 times independently. Untreated cells were used as negative control. WST1 (Sigma-Aldrich) was added for 2 h, and absorbance of produced formazan was measured using CLARIOstar (BMG Labtech). After calculating treosulfan half-maximal inhibitory concentration (IC_50_) for each cell line, experiments were repeated using treosulfan IC_50_ and different fludarabine concentrations (1–1000 µM).

Tumor spheroids were generated by culturing 10e5 cells for 72 h in 96-Well Nunclon Sphera-Treated U-Bottom Microplates (Thermo Fisher), using corresponding IC_50_:50 µM treosulfan, 50 µM fludarabine, or 50 µM treosulfan + 25 µM fludarabine were then added for 48 h. Spheroids were dissociated by pipetting, and cell viability was measured using WST1.

### Effect of cytostatics on MSLN expression

MSLN + SKOV3 and OVCAR4 (0.15e6) cells were incubated with treosulfan or fludarabine at a concentration corresponding to half of IC_50_ (in normoxic conditions, see Table 1), and MSLN expression was measured after 48 h by flow cytometry (CytoFLEX, Beckman Coulter). Untreated cells served as negative control. Results were analyzed using FlowJo Software (BD Life Sciences).

**Table 1:** IC_50_ of treosulfan and fludarabine on SKOV3 and OVCAR4 cells

Tumor cell line	Cytostatic	Normoxia		Hypoxia	
		Parental	MSLN+	Parental	MSLN+
SKOV3	Treosulfan	47.69 µM**	51.50 µM	135.0 µM**	100.8 µM
	Fludarabine	45.94 µM****	49.28 µM****	60.25 µM	62.05 µM
	Fludarabine seria with Treosulfan IC_50_	27.99 µM**** (Treosulfan 50 µM)	14.55 µM**** (Treosulfan 50 µM)	32.37 µM^####^ (Treosulfan 135 µM)	5.42 µM^####^ (Treosulfan 100 µM)
OVCAR4	Treosulfan	53.84 µM^***^	58.82 µM^****^	179.0 µM^***^	204.6 µM^****^
	Fludarabine	21.43 µM	27.88 µM	11.25 µM	19.73 µM
	Fludarabine seria with Treosulfan IC_50_	9.90 µM^####^ (Treosulfan 55 µM)	8.76 µM **** (Treosulfan 55 µM)	4.36 µM^####^ (Treosulfan 180 µM)	3.66 µM**** (Treosulfan 205 µM)

**p = 0.005, ***p = 0.001, **** and ^####^*p* < 0.0001

### T cell apoptosis assessment

PBMCs (3e5 cells) were incubated for 24 h with treosulfan or fludarabine (10 µM). Cells were stained with Annexin V-APC antibody and 7-AAD in Annexin Binding Buffer (BD Biosciences), and apoptosis was measured by flow cytometric analysis (CytoFLEX).

### T cell functions and mitochondria assessment

MSLN-CAR + T cells were incubated for 24 h with cytostatics. Afterward, cells were stimulated for 6 h, in either normoxia or hypoxia, with MSLN + OVCAR3 cells (effector:target ratio 1:1) in the presence of BFA (Sigma-Aldrich), GolgiStop (BD Biosciences), and anti-CD107a antibody. After 6 h, cells were stained with anti-CD3, -CD4, and -CD8 antibodies, fixed and permeabilized (BD Cytofix/Cytoperm kit, BD Biosciences), stained with LIVE/DEAD Fixable Aqua Dead Cell, anti-IL-2, -TNF, and -IFN*γ* antibodies (Supplementary Table S1). T cells mitochondria were evaluated by flow cytometric analysis using: tetramethylrhodamine methyl ester (TMRE), MitoSOX, or MitoTracker green (Invitrogen).

To measure killing, M28z CAR + T cells were incubated for 24 h with cytostatics, then stimulated for 24 h in normoxia or hypoxia, with MSLN + OVCAR3 cells (effector:target ratio 1:1). Matched untransduced (CAR-) untreated T cells served as negative control. OVCAR3 tumor cells (modified to express luciferase) killing was measured by bioluminescence using One-Glo Luciferase Assay (Promega) and reading with CLARIOstar multireader. M28z CAR T cells-specific killing was calculated as follows:

$${1}00\% {\text{ specific killing}} = {1}00 \times \frac{{\text{OVCAR3 bioluminescence after incubation with M28zCAR T - cells}}}{{\text{OVCAR3 bioluminescence after incubation with control T - cells}}}$$

### Data and statistical analysis

Viability of treated cells (WST1 assay) was expressed as percentage of untreated cells viability. Ovarian cancer cells IC_50s_ was calculated by four-parameter logistic regression model, comparison done using Tukey’s multiple comparison test two-way ANOVA, GraphPad Prism (GraphPad software).

## Results

### Hypoxia and MSLN expression modulate differently the cytotoxicity of treosulfan and fludarabine against SKOV3 and OVCAR4 tumor cells

**Fig. 1:**
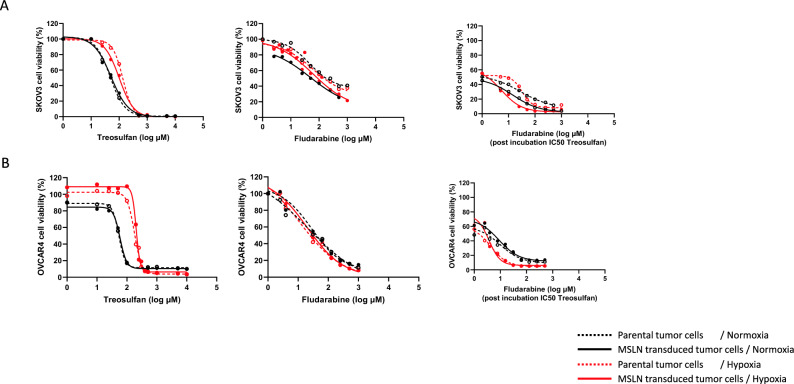
IC_50_ determination of treosulfan and fludarabine on SKOV3 and OVCAR4 cells. **A** IC_50_ determination of treosulfan, fludarabine, and fludarabine when combined with treosulfan (at determined IC50 concentration in respective condition) on parental, MSLN-transduced SKOV3 cells in normoxia (21% O) or hypoxia (2% O_2_). **B** IC_50_ determination of treosulfan, fludarabine, and fludarabine when combined with treosulfan (at determined IC50 concentration in respective condition) on parental, MSLN-transduced OVCAR4 cells in normoxia (21% O_2_) or hypoxia (2% O_2_). Solid lines represent MSLN-transduced cells, dotted lines represent parental tumor cells, black lines represents normoxic conditions, and red lines represent hypoxic conditions. Each point represents the mean of 3–5 independent experiments performed in triplicates

We evaluated treosulfan and fludarabine IC_50_ against SKOV3 and OVCAR4 cells (parental and MSLN+) in normoxia and hypoxia. Treosulfan IC_50_ for parental and MSLN + SKOV3 cells were comparable in normoxia and hypoxia (Table 1 and Fig. 1A), and hypoxic incubation induced a significant increase in IC_50_ for parental SKOV3 cells (*p* = 0.005). This impact of hypoxia was not seen for fludarabine IC_50_ where a difference (*p* < 0.0001) was only seen between parental and MSLN + SKOV3 cells in normoxia. When co-incubated with treosulfan, fludarabine IC_50_ was significantly lower (*p* < 0.0001) for MSLN + SKOV3 cells compared to parental cells in normoxia and hypoxia.

With OVCAR4 cells (Table 1 and Fig. 1B), treosulfan IC_50_ was significantly increased (*p* < 0.0001) in hypoxia both for parental and for MSLN + cells. No differences between the different conditions were observed for fludarabine IC_50_, but when co-incubated with treosulfan, fludarabine IC_50_ was significantly decreased (*p* < 0.0001) in hypoxia for parental and MSLN + OVCAR4 cells.

Hypoxia appears to impact the IC_50_ of treosulfan and combination of treosulfan and fludarabine on ovarian tumor cells. This impact was cell line-dependent: More differences were observed with (parental and MSLN+) OVCAR4 cells. Hypoxia increased treosulfan IC_50_ for SKOV3 and OVCAR4 cells but lowered fludarabine IC_50_ when combined with treosulfan for OVCAR4 cells.

MSLN expression modulated SKOV3 cells (but not OVCAR4 cells) drug resistance. When SKOV3 overexpressed MSLN, fludarabine IC_50_ was increased in normoxia but decreased when co-incubated with treosulfan in normoxia and hypoxia.

**Fig. 2:**
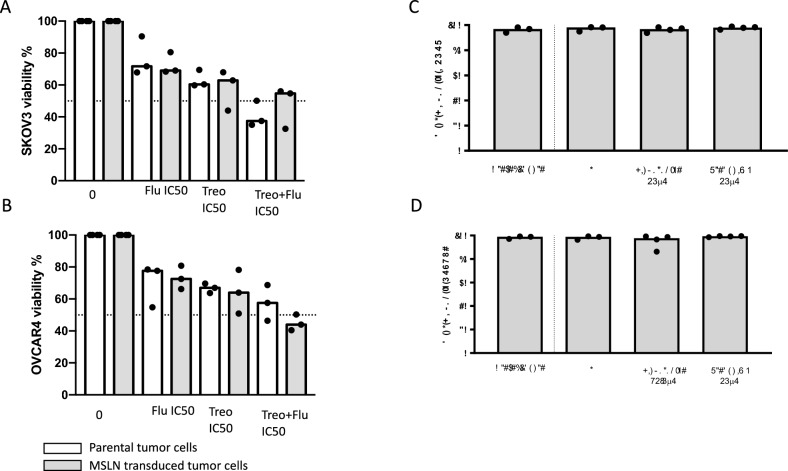
Impact of treosulfan and fludarabine on spheroids tumor cells and on MSLN cell surface expression. **A** Viability of SKOV3 (parental or MSLN-transduced) spheroids cells after incubation for 48 h with treosulfan (50 µM), fludarabine (50 µM), or combination of fludarabine (25 µM) and treosulfan (50 µM) (*n* = 3). **B** Viability of OVCAR4 (parental or MSLN-transduced) spheroids cells after incubation for 48 h with treosulfan (50 µM), fludarabine (50 µM), or combination of fludarabine (25 µM) and treosulfan (50 µM) (*n* = 3). White bars: parental tumor cells; gray bars: MSLN-transduced tumor cells. **C** MSLN cell surface expression of MSLN-transduced SKOV3 cells after incubation for 48 h with treosulfan (25 µM) or fludarabine (25 µM) (*n* = 3). **D** MSLN cell surface expression of MSLN-transduced OVCAR4 cells after incubation for 48 h with treosulfan (25 µM) or fludarabine (25 µM) (*n* = 3). Exposure without drugs was used as negative control

Culturing tumor spheroids with single cytostatics increased their resistance to cytostatics (Fig. 2A, B). Similar trends were observed for SKOV3 and OVCAR4, and the median frequency of live tumor cells (parental and MSLN+, SKOV3, and OVCAR4 cells) retrieved from the spheroids after incubation with treosulfan was between 69–78% and between 60–67% with fludarabine, but was lower (37–58% live tumor cells) when combination of both cytostatics was used.

In addition, MSLN + SKOV3 and OVCAR4 cells incubation with treosulfan and fludarabine for 48 h showed that the frequency (Fig. 2C, D) of MSLN+ expression was unchanged (≥ 95%) compared to preincubation or control.

### Treosulfan or fludarabine does not impact MSLN-CAR T cells

**Fig. 3:**
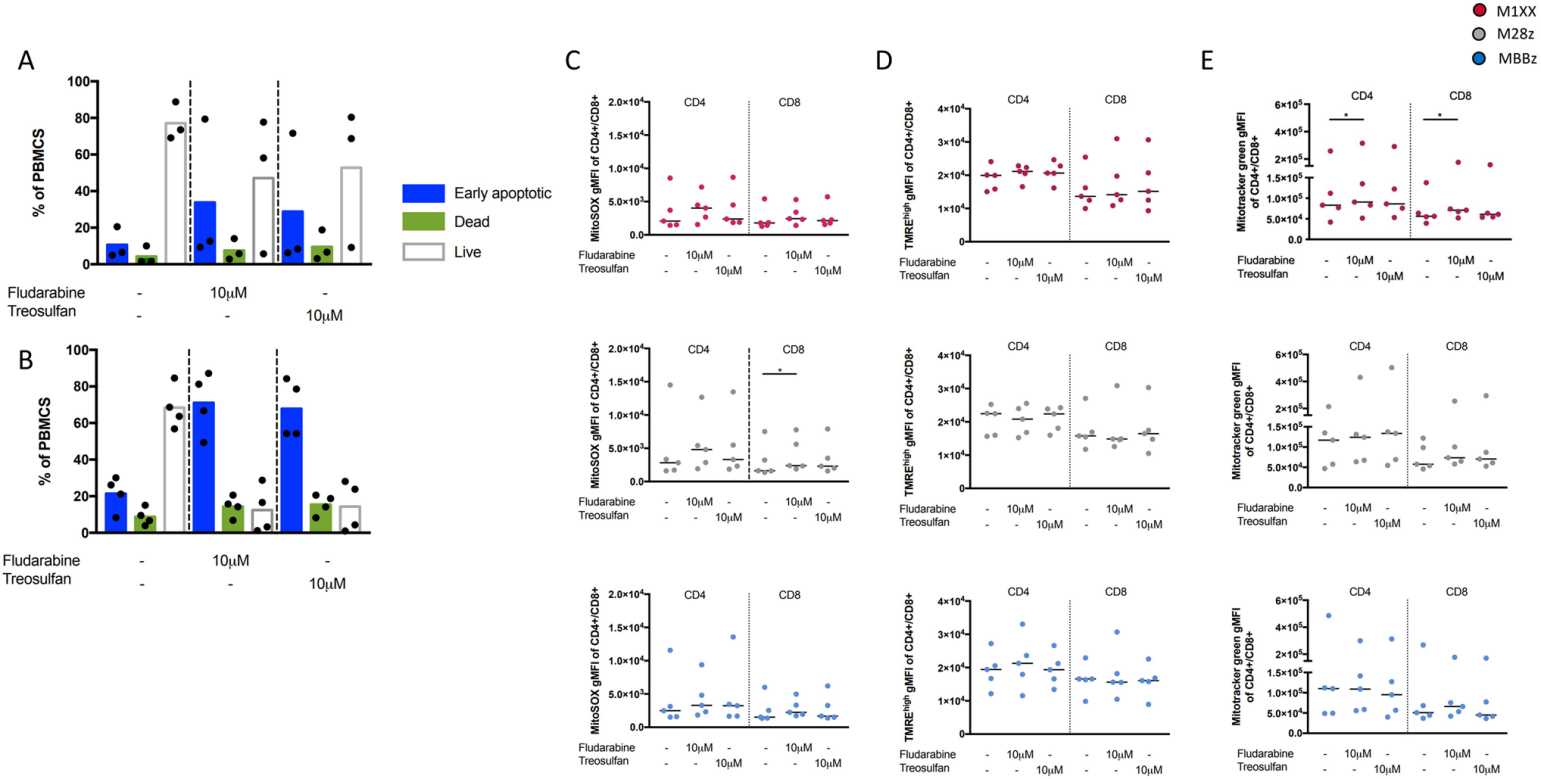
Treosulfan and fludarabine do not impact MSLN-CAR T cells mitochondrial functions. **A**, **B** The impact of treosulfan (10 µM) or fludarabine (10 µM) on the viability of healthy donors PBMCs was assessed after 24 h (**A**) and 48 h (**B**) by flow cytometry after Annexin V and 7-AAD staining. Blue: early apoptotic, green: dead, and white: live cells (*n* = 3). **C**–**E** Healthy donor MSLN-CAR T cells were exposed for 24 h to treosulfan (10 µM) or fludarabine (10 µM), and mitochondrial ROS (gMFI of MitoSOX + T cells **C**), mitochondrial membrane potential (gMFI of TMRE^high^ T cells **D**), and mitochondrial mass (gMFI of MitoTracker Green + T cells **E**) in CD4+ and CD8+ T cells were determined by flow cytometry lls (*n* = 5). gMFI; geometric median fluorescence intensity. Red: M1XX MSLN-CAR T cells, gray: M28z MSLN-CAR T cells, and blue: MBBz MSLN-CAR T cells. Friedman and Dunn’s post-hoc tests, *p < 0.05

We tested the impact of treosulfan or fludarabine on PBMCs viability. Incubation with 10µM treosulfan or fludarabine led to ~ 50% (median, with inter-individual variability) and ~ 12% (median) cell viability after 24 h and 48 h, respectively (Fig. 3A, B). We assessed the impact of 10µM treosulfan and fludarabine on surviving CAR T cells and measured mitochondrial reactive oxygen species (ROS) production, mitochondrial membrane potential, and mitochondrial mass by using MitoSOX, TMRE, and MitoTracker Green dyes, respectively, in different MSLN-CAR T cell products (M28z, MBBz, and M1XX). Incubation for 24 h with treosulfan or fludarabine did not impact the mitochondrial markers regardless of the MSLN-CAR construct or T cell (CD4+ and CD8+) subset. Significant differences were detected in the presence of fludarabine: increased ROS production in CD8 + M28z CAR T cell product and mitochondrial mass of CD4+ and CD8+ in the M1XX CAR T cell product (Fig. 3D).

**Fig. 4:**
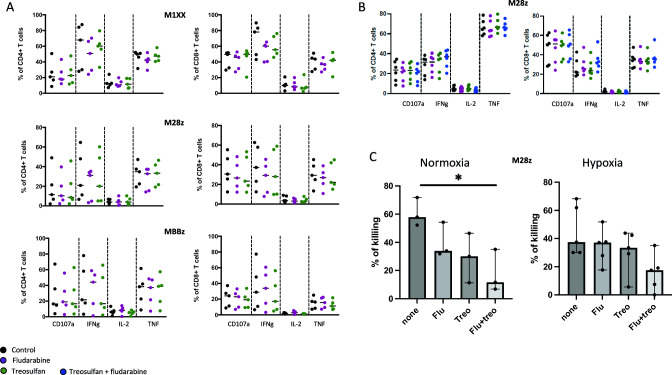
Treosulfan and fludarabine do not impact MSLN-CAR T cells effector functions. Healthy donors MSLN-CAR T cells were exposed for 24 h to treosulfan (10 µM), fludarabine (10 µM), or combination of treosulfan (10 µM) and fludarabine (10 µM). For cytokine and degranulation measurements, drug-exposed MSLN-CAR T cells were stimulated for 6 h with MSLN + OVCAR3 tumor cells in normoxic (**A**) or hypoxic (**B**) conditions. Expression of cytokines (IFN*γ*, IL-2, and TNF) and degranulation marker (CD107a) were measured by flow cytometry. Exposure without drugs was used as negative control. **A** Frequency of positive cells in CD4+ (left) and CD8+ (right) T cells in M1XX (top), M28z (middle), and MBBz (bottom) MSLN-CAR T cells (*n* = 5). Medians are presented. Friedman test was performed (no significant differences were observed). **B** Frequency of positive cells in CD4+ (left) and CD8+ (right) T cells in M28z CAR T cells (*n* = 6). Medians are presented. Friedman test was performed (no significant differences were observed). **C** For killing measurement, drug-exposed M28z MSLN-CAR T cells were stimulated for 24 h with MSLN + OVCAR3 tumor cells in normoxic (left panel) or hypoxic (right panel) conditions (*n* = 3–5). Medians and range are presented. Friedman test was performed, *p < 0.05

Similarly, 24-h exposure to treosulfan or fludarabine (10 µM) did not impact MSLN-CAR T cells CD107a expression, cytokine production, or tumor killing when co-incubated with MSLN+ target T cells in normoxia or hypoxia (Fig. 4A–C). However, combined exposure to fludarabine and treosulfan significantly decreased tumor killing in normoxia but not in hypoxia (Fig. 4C).

## Discussion

Treosulfan and fludarabine conditioning regimen is widely used for various malignancies. We evaluated in vitro treosulfan and fludarabine (and their combination) as potential conditioning prior to MLSN CAR T cell therapy for ovarian cancer treatment.

We showed that treosulfan and fludarabine mediated the killing and, in combination, had a synergetic effect on SKOV3 and OVCAR4 ovarian cancer cell lines. Since solid tumors form hypoxic niches, we tested the two drugs in normoxic and hypoxic conditions. Hypoxia increased SKOV3 and OVCAR4 resistance to treosulfan, but not fludarabine. Interestingly, when treosulfan and fludarabine were combined, tumor cells drug sensitivity was increased, and a similar trend was observed when using a tumor spheroid model (where lower oxygen levels would be expected). MSLN expression has been shown to promote resistance to some drugs (e.g., platinum and cyclophosphamide combination) [16]. MSLN overexpression was previously reported to induce resistance to treatment in pancreatic cancer cells [17]. Overexpression of MSLN appeared to modulate sensitivity of SKOV3 cells to fludarabine (increasing it when alone, decreasing when combined with fludarabine), but this was not seen for OVCAR4 cells. Our results highlight the importance of testing relevant tumor cells (and associated antigens of interest) and conditions (e.g., hypoxia) when assessing the cytotoxic effect of cytostatics drugs.

Exposure to treosulfan and fludarabine did not impact MSLN cell surface expression on SKOV3 and OVCAR4 suggesting that these two drugs do not negatively impact this tumor antigen expression and, subsequently, the capacity for MSLN-CAR T cells to bind MLSN and kill tumor cells.

Lymphodepleting conditioning regimens are usually given within a week before CAR T cell treatment [18], and CAR T cells are given within two days after the last cytostatic infusion in order to avoid negative interference. Previous reports have shown that exposure for 24 h with 1µM fludarabine (followed by six days of culture) increased the proliferation and frequency of HDs memory T cells [19]. Exposure to treosulfan and fludarabine did not impact MSLN-CAR T cells effectors functions (degranulation and cytokine production) or mitochondrial defects, while combination of treosulfan and fludarabine decreased MSLN-CAR T cells anti-tumor killing in normoxia but not in hypoxia. Our results indicate that even in combination, these two drugs do not negatively impact MSLN-CAR T cells functions in the context of anhypoxic solid tumor.

Altogether, our study shows that treosulfan and fludarabine can mediate cytotoxicity toward SKOV3 and OVCAR4 ovarian cancer cell lines without inducing loss of MSLN cell surface expression or having a negative impact on MSLN-CAR T cells. Further preclinical work is needed to determine the optimal dose and timing for preconditioning with treosulfan and fludarabine prior to MSLN-CAR T cell therapy for the treatment of ovarian cancer.

## Supplementary Information

Below is the link to the electronic supplementary material.

Supplementary file1 (DOCX 28 kb)
