# Supplementary material for: Impact of fludarabine and treosulfan on ovarian tumor cells and mesothelin chimeric antigen receptor T cells
Source: Cancer Immunol Immunother. 2024 Jul 2;73(9):163. doi: 10.1007/s00262-024-03740-3 (PMC11219644; doi:10.1007/s00262-024-03740-3)
Supplement: Supplementary file 1 — Supplementary file1 (DOCX 28 kb) [file 262_2024_3740_MOESM1_ESM.docx]

**Supplementary Material**

**Table S1.** **Antibodies for cells intra-, extra-cellular and mitochondrial stainings**

| Antibodies | Vendor |
| --- | --- |
| Alexa Fluor® 647 anti human IFN-γ monoclonal antibody | BD Pharmingen™ |
| Alexa Fluor® 700 anti human IL-2 monoclonal antibody | BioLegend® |
| Allophycocyanin (APC) anti-human IFN-γ monoclonal antibody | BD Pharmingen™ |
| R-phycoerythrin (PE)-eFluor® 610 anti human TNF-α monoclonal antibody | Invitrogen |
| Alexa Fluor® 700 anti human CD3 monoclonal antibody | BD Pharmingen™ |
| APC anti-human CD3 monoclonal antibody | BD Pharmingen™ |
| APC anti-human Mesothelin monoclonal antibody | R&D systems |
| Allophycocyanin-Cyanine 7 APC-Cy™7 anti-human CD8 monoclonal antibody | BD Pharmingen™ |
| Brilliant Violet 510™ anti-human CD4 monoclonal antibody | BioLedgend® |
| FITC anti-human CD4 monoclonal antibody | BD Pharmingen™ |
| PE anti-human CD107a monoclonal antibody | BD Pharmingen™ |
| PE-cyanine7 (PE-Cy™7) anti human CD3 monoclonal antibody | BD Pharmingen™ |
| LIVE/DEAD™ Fixable Aqua Dead Cell Stain Kit,  for 405 nm excitation | Invitrogen™ |
| APC Annexin V | BD Pharmingen™ |
| 7-Aminoactinomycin D (7-AAD) | BD Pharmingen™ |
| MitoSOX™ Red Mitochondrial Superoxide Indicator | Invitrogen™ |
| MitoTracker™ Green FM | Invitrogen™ |
| Tetramethylrhodamine, ethyl ester, perchlorate (TMRE) | Invitrogen™ |
